# Low-Coherence Interferometric Fiber Optic Sensor for Humidity Monitoring Based on Nafion^®^ Thin Film †

**DOI:** 10.3390/s19030629

**Published:** 2019-02-02

**Authors:** Erwin Maciak

**Affiliations:** Department of Optoelectronics, Faculty of Electrical Engineering, Silesian University of Technology, 2 Krzywoustego Str., PL44-100 Gliwice, Poland; erwin.maciak@polsl.pl; Tel.: +48-32-237-2182

**Keywords:** fiber optic humidity sensor, polymer Nafion^®^ thin film, low-coherence interferometry, layered Fabry-Perot interferometer, polymer dip coating technology

## Abstract

The main aim of this work was the design and development simple fiber optic Fabry-Perot interferometer (FPI) sensor devices for relative humidity (RH) sensing with emphasis on high sensitivity and good stability. The RH fiber FPI sensor is fabricated by coating the end of a cleaved standard multi-mode (MM) fiber with hydrophilic Nafion^®^ sensing film. The Nafion^®^ thin film acts as an active resonance cavity of the low-coherence interferometric sensing structure. The fringe pattern, which is caused by interfering light beam in the Nafion^®^ thin film will shift as the RH changes because the water molecules will swell the Nafion^®^ film and thus change optical pathlength of the sensing structure. The operating principle of a FPI sensor based on the adsorption and desorption of water vapour in the Nafion^®^ and the limitations of this sensor type are discussed in this work. The fiber optic hygrometer was tested in the visible (400–900 nm) region of spectra for measurement of relative humidity (RH) in the range of 5.5–80% at room temperature (RT) in air. The fiber optic humidity sensor has a very short response time (t_90_ = 5–80 s) and a fast regeneration time (t_10_ = 5–12 s) as good as commercial sensors.

## 1. Introduction

Advanced technology, which is a powerful force in the development of civilization, requires finding effective sensor techniques for measuring the concentrations of various substances. In global industry, the problems connected with monitoring the concentration of various gas analytes (e.g., toxic, biological, industrial and others) have become particularly important due to environmental protection needs. High-tech sensor applications, including detection, measurement and control systems for pollutants and gas emissions, are extremely important for an ecologically sustainable development. Information about the humidity level present in an ambient environment or in industry processes is one of the most important. Despite the large number of sensor techniques and materials investigated, it is not easy to identify cheap and reliable method for humidity sensor construction that can measure a wide range of relative humidity (RH) values [1,2,3,4,5].

Humidity measurements determine the amount of water vapor present in a gas that can be a mixture, such as air. The importance of the humidity measurements in science and industry arises from the fact that many important natural and synthetic materials change their characteristics with moisture. The amount of water vapor present in a gas can be measured as RH, dew point (DP), and parts per million (ppm) [1,2,3]. In electronic humidity sensors, the most commonly used units for moisture level measurements are RH, which is expressed as a percentage. RH is defined as the amount of moisture in a gas (this gas is usually air) compared to what a gas can contain at given temperature. When the air can’t hold all the moisture, then it condenses as dew (liquid phase). Thus, relative humidity is expressed as the ratio of the partial pressure of water vapor (*p_w_*) present in a gas to the saturation vapor pressure (*p_s_*) of a gas at a given temperature as in Equation (1):

(1) $$RH=\frac{p_{w}}{p_{s}}\times 100\%.$$

RH is a significant function of temperature.

Water vapor is a substance encountered in our daily lives and in many areas of industry. Thus, humidity measurements are an extremely important in the chemical processing, electronics, food, pharmaceutical, automotive, agriculture, and many other industries [1,2,3,4,5]. The production and storage of different materials can also be also improved with better control of humidity [1,2,3]. Depending on the operating conditions moisture levels can be measured by various types of sensors based on a variety of water vapor sensing materials and sensor technologies. Chen and Lu [1], and Rittersma [2] have presented broad reviews of humidity sensors for a wide variety of applications. The major scientific and industrial application concerns electronic humidity sensors with electric output [2], but like other electronic sensors, electronic RH sensors are susceptible to electromagnetic interference. Compared with their electronic counterparts, fiber optic sensors have a number of advantages. Yeo et al. [4] and Ascorbe et al. [5] have presented reviews of various fiber optics-based humidity sensing techniques. Particular attention should be given to the design of fiber humidity sensors based on etched polymer fiber optics (POFs) [6,7]. Rajan et al. [6] demonstrated that FBGs produced in etched polymer fiber optics (POFs) present enhanced humidity sensitivity compared to pristine fibers. Etched POF gratings were fabricated by solvent etching after the grating inscription, which resulted in very short response times for humidity sensors based on POF. Another solution is a sensor based on the variations in the etched stress-POF material’s properties due to ambient humidity variations [7]. The stress on the etched fibers leads to output power variations due to the effective stress-optic effect. The water vapor intake into the etched core of PMMA stress-optic polymer fibers causes fiber refractive index changes [7]. These sensors have short response times and have the potential for low cost fabrication and applications. However, the remaining diameter is so thin that such etched POFs are not convenient to handle and are very fragile.

An advantage of fiber optic sensors over conventional electronic humidity sensors is that they are able to operate with excellent environmental resistance. Fiber optic sensors are unaffected by electrical noise. Furthermore, these fiber sensors, due to their extremely compact sensor head and less weight, allow for easy installation in limited measurement spaces. Fiber optic sensor technologies have the potential for multi-gas detection using different sensor interrogation techniques such as intensity, wavelength, multi-color detection, phase and polarization of the output light response signals. Intrinsic safety due to removal of electrical power from measurement areas, high sensitivity, wide dynamic range, and the possibility of multipoint detection with potential of full distributed sensing are undoubtedly some of the most important advantages of fiber optic gas sensors. For these reasons, fiber optic sensors have been explored in optical sensing devices and systems in the last several decades and numerous achievements have been realized in the chemical sensing and biosensing fields [4,5,6,7,8,9,10].

Recent research on developing suitable and cost effective sensors for humidity measurement has focused on sensors based on inorganic materials, especially ceramic and semiconductor materials [10], and organic materials [11,12]. Researchers have developed different sensing structures and types of fiber optic humidity sensors, that are based on various physical effects. The use of phenomena such as interferometry [10,12,13], optical evanescent wave [14,15], surface plasmon resonance (SPR) [15,16], in-fibre gratings: Bragg (FBG) [6,17,18,19] and long period (LPG) [20], hetero-core fibers [21], and application photonic fibers [22] in humidity fiber optics and optoelectronics sensors have been proposed and demonstrated. Furthermore, RH optical measurement techniques include direct spectroscopic, the color change in the sensing materials with humidity, changes of dimensions, refractive index, or reflectivity of optical materials [1,4,5,7,8]. Optical humidity sensors have also been reported, based on materials whose optical absorption or fluorescence intensity changes on exposure to humidity [2,4,5].

The main aim of the research presented in this paper was to describe a low-cost manufacturing method and an extremely simple type of an optoelectronics humidity sensor compatible with commercial Fabry-Pérot (*FP*) white-light interferometry technology. Low-coherent fiber-optic interferometric humidity sensor is based on Nafion^®^ thin films. A Nafion^®^ thin film acts as an optically active resonance cavity of the interferometric sensing structure. The operating principle of a FPI humidity sensor based on the method with spectral and intensity analysis of reflection interferogram is discussed. The fringe pattern, which is caused by interfering light beam in the Nafion^®^ thin film will shift as the RH changes because the water molecules will swell the Nafion^®^ film and thus change optical pathlength of the sensing structure. The results from this study show optical humidity sensing combined with relatively cheap optical components and instruments (e.g., multi-mode fiber optics, light sources, photodetectors, etc.) that were developed for use in the visible region (350–780 nm). This is of extremely significant importance on the cost of the sensing device. The reported results are of interest to present FPI sensor devices as candidates for possible commercial utilisation. The main advantages are low cost, stability and large variation of the optical signals, both spectral and in intensity mode.

## 2. Materials and Experimental Methods

### 2.1. Nafion^®^-Based Humidity Sensing Material

Nafion^®^ is special material that possesses widespread applications in the area of electrochemistry analysis, chemical sensor, and nanomaterials [11,12,23,24,25,26,27]. Nafion^®^ is material whose optical parameters (refractive indices, thickness, etc.) are reversibly modified by specific gases, such as water molecules, alcohol vapor, and ammonia gas. Thus, it has a considerable potential for use as optochemical sensing material for fiber optic gas sensors. In this paper, Nafion^®^ was selected as a sensing film for the RH sensor. Also, Nafion^®^ is a good cation exchanger. This material offers many advantages, such as good thermal stability, very good chemical inertness (excluding polar substances i.e., alcohols, ammonia gas, etc.), good permeability to polar analytes, and mechanical strength. For these reasons, the Nafion^®^ is widely used the proton exchange membrane (PEM) type fuel cells and industrial gas dryers [23,24,25,26,27,28]. Three structural areas can be identified and distinguished in Nafion^®^: the hydrophobic fluorocarbon backbone, hydrophilic ionic clusters of sulfonic acid groups, and an interfacial region. It is these ionic clusters which are responsible for the physisorption of polar water molecules [28,29,30,31,32].

### 2.2. Sensing Principle

**Figure 1:**
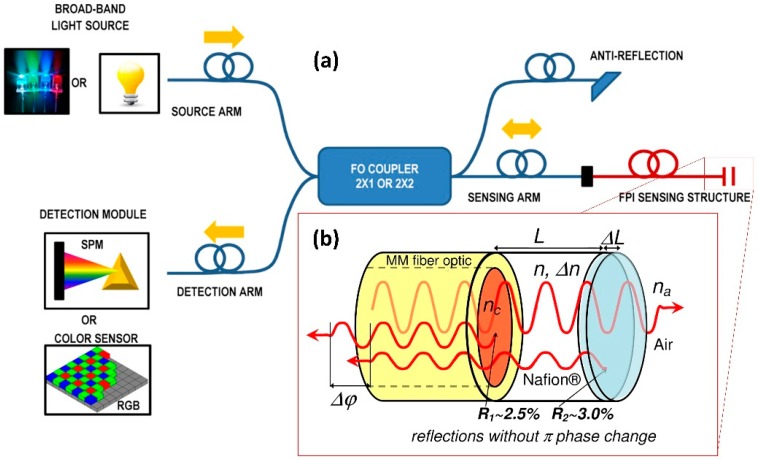
(**a**) Schematic presentation of the concept of the low-coherence (white-light) interferometry FP sensors. (**b**) Schematic representation of the FP humidity fiber optic sensor operation principle. Upon moisture absorption Nafion^®^ thin film (resonance cavity of the FP sensor) expansion deforms the interferometer, inducing a shift of its fringe pattern. Refractive index changes in the Nafion^®^, upon moisture absorption, also contribute to this shift.

Fiber Fabry-Pérot interferometers (FPIs) have been applied to the sensing of temperature, strain, as chemical gas sensors, and for measuring the ultrasonic pressure in composite materials [8,9,12,13]. This early work laid the foundation for extensive research and development as well as commercialization. Fiber FPIs are extremely sensitive to perturbations that affect the optical path length between the two mirrors. The sensing region can be very compact (hundreds of nanometers). An FP sensor cavity can be interrogated with a variety of optical signal measurement methods. The most robust and reliable way is low-coherence interferometry methods [33,34,35,36,37,38,39,40]. Figure 1a shows the concept of low-coherence (white-light) interferometry interrogation in FP sensors. FP low-coherence interferometry is essentially a method used to examine the interferometric cavity at different wavelengths over a certain spectral range [8,12,33,34,35]. In general, fiber FPI transducer/sensor architecture is always made with similar optical and optoelectronics parts, including a light source, a fiber optic track consisting of a Y or X shaped coupler, an optical sensing element (fiber optic with an opto-physicochemical sensing structure), and a photodetector with a recording device. The selectivity, sensitivity and time response of the sensor can be tailored by a proper choice spectral range and changing the optical configuration of interferometric sensing structure (e.g., number of layers, appropriate film thicknesses, etc.). In cases when the physicochemical changes of the sensing structure are accompanied by changes in the optical properties (e.g., refractive index, extinction, film thickness changes) they play a role in optical sensing. As an effect of the changes of optical parameters of the sensor layer(s), an optical response can easily be obtained directly using spectral or intensity signal processing, as in this case. To obtain a highly interfering signal from the FP cavity, the optical path difference (distance) (OPD) between different beams has to be much smaller than the coherence length of the light. If the OPD of the interferometer is larger than the coherence length of the light, the reflected beams will not effectively interfere with each other to generate fringes. Thus, the thickness of the Nafion^®^ layer which is the resonance cavity of the interferometer should be on the order of several hundred nanometers. The OPD between the two reflectors is expressed by Equation (2):

*OPD* = 2*nL* (2)

where *n* is the refractive index and *L* is the thickness film (distance between the two reflectors) of the Nafion^®^ at a certain wavelength.

As shown in Figure 1b, a fiber FPI RH sensor consists of two mirrors having reflectance of *R*_1_ and *R*_2_ respectively, separated by a cavity of a certain length. In the presented sensing structure of the fiber RH probe first mirror (from the fiber) of the interferometer is realized on the boundary multi-mode (MM) fiber/Nafion^®^, because this interface has a dielectric contrast (*n_c_* = 1.48 RIU (refractive index units)/*n* = 1.37 RIU at *λ* = 589.3 nm) I calculated the reflectance of this mirror about *R*_1_ = 2.5%. The Nafion^®^ layer is the resonance cavity of the interferometer and by adjusting the thickness and affecting the optical properties it becomes possible to tune the interferometer within a wide range. The second mirror (*R*_2_ = 3.0%) is the Nafion^®^/ambient air (*n* = 1.37 RIU/ *n*_a_ = 1.00 RIU at *λ* = 589.3 nm) interface.

The reflectance at both the MM fiber end/Nafion^®^ and the Nafion^®^/air interfaces of the RH sensor probe are low (about 3.0%), and the response is a periodic function similar to a two-beam interferometer. The finesse of such an interferometer is low. Due to the low reflectance at each reflector, the interference between the two reflections at wavelength *λ* is approximately described by Equation (3) [8,33,39]:

(3) $$I\left(\lambda \right)=I_{0}\left(\lambda \right)\left[R_{1}+R_{2}+2\sqrt{R_{1}R_{2}}\cos\left(\Delta \varphi \right)\right]$$

where *I*(*λ*)—reflected light power measured at wavelength *λ*; *I*_0_(*λ*)—power of the light source at wavelength *λ*; *R*_1_, *R*_2_—reflectivity of the reflectors at wavelength *λ*; and Δ*φ*—the phase difference between the two light beams reflected from the mirrors of the sensing interferometer, Δ*φ* can be written as Equation (4):

(4) $$\Delta \varphi =\frac{2\pi OPD}{\lambda }=\frac{4\pi nL}{\lambda }.$$

As a consequence, the interference signal is a function of the effective cavity length. The humidity of the air induces OPD changes and modulates the optical reflected signal. By monitoring the sensor output, the applied humidity can be measured.

FPI sensors based on multimode (MM) fibers are much less sensitive to the angular orientation of the interferometric structure and any optical distortions between the cavity and the fiber than single mode solutions. The proposed FPI sensor with multimode illuminating and receiving fiber exhibits low sensitivity to the optical quality of cavity and its angular orientation (flat parallelism). Of course, it is expected existing intermodal interference in MM fiber optics. But the path length distributions measured in cavity suggest that the effect of intermodal dispersion is very weak and the effect of additional satellite peaks that may result from the intermodal dispersion are not evident. Thus the intermodal coupling in the fibres does not significantly limit the performance of multimode FPI. Moreover, the literature [41,42,43] shows that the intermodal coupling inside the multimode fibers does not have a significant effect to performance of multimode FPI structure [42] and does not lead to the destruction of interferogram. On the other hand, practical industrial applications of MM fiber optical interferometry for in-line measurement of geometric parameters are easier to implement due to coupling broadband sources and detectors to MM fibers.

Upon water vapor absorption, generally two factors can cause shifts of the interferometer reflectance spectrum (fringe pattern). First, as depicted in Figure 1b, Nafion^®^ swelling deforms the interferometer, resulting in an increase (Δ*L*) of the mirror separation (*L*). This mechanism is the dominant one [27,28,29,32]. Secondly, the refractive index of the Nafion^®^ sensing coating (*n*) should change by a factor Δ*n* upon moisture absorption. Due to moisture absorption refractive index of the Nafion^®^ film decreases, thus Δ*n* is negative. Those effects change the interferometer *OPD*, and contribute to shift the measured reflectance spectrum of the sensing structure. The total *OPD* change of the *FP* humidity sensor can be given as Equation (5):

(5) $$\Delta OPD=2\left(-\Delta nL+n\Delta L\right)=OPD\left(-\frac{\Delta n}{n}+\frac{\Delta L}{L}\right).$$

### 2.3. Sensor Fabrication

**Figure 2:**
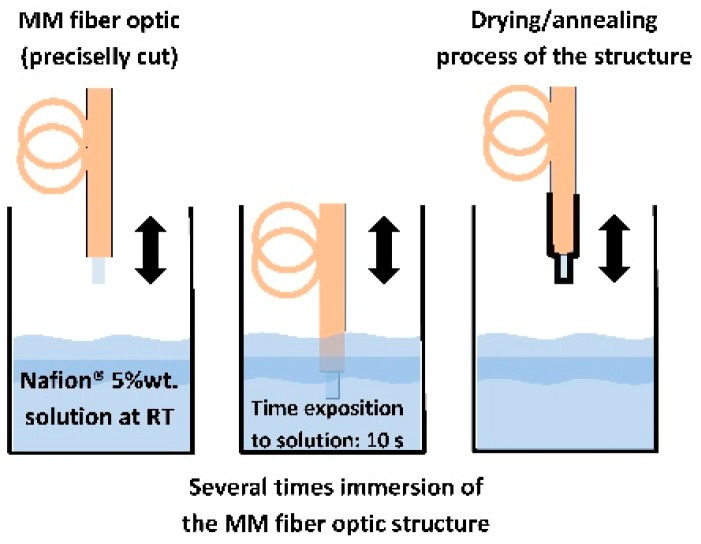
Technology of deposition of Nafion^®^ sensing thin film.

The fiber sensor probe is made with a multi-mode (MM) gradient index (GI) fibers. Dip (or immersion) coating is a relatively simple technique for coating glass and plastic substrates with uniform thin films from liquid phase. This work employs dip coating to deposit Nafion^®^ layer onto end of the fiber optic substrate as uniform sensing coating. The Nafion^®^-based sensing structures was fabricated by the following approach. Nafion^®^ perfluorinated ion-exchange resin solution (5 wt.% in mixture of lower aliphatic alcohols and water, contains 45% water, cat. 510211) was purchased from Sigma Aldrich (Poznań, Poland) and used without any further purification. It was immobilized directly onto the end of precisely cleaved MM fiber optic (62.5/125 µm) by dip coating at room temperature for 10 s–Figure 2. After the coating process, the coated fiber optics were then dried for 120 min. at 40 °C, and cured for 15 min. at temperature of 110 °C. After solvent evaporation, the interfacial Nafion^®^ thin film is about several hundred nanometers thick. After deposition of the first layer of about one hundred nanometers thickness, the fiber optic substrate becomes more adhesive and the next layers will be deposited at the immersion process are thicker than first. Subsequently, a Nafion^®^-based sensing structure will be built up by successive depositions of single layers. As a result of the single process deposition a Nafion^®^ layer of a thickness of approximately 260 nm (±20 nm) was obtained. In order to obtain thicker layers, the deposition process must be repeated several times. The accuracy of the layer thickness of the single process deposition is about 10%. In the present study sensing structures fabricated with two layers of Nafion^®^ have been used.

As mentioned above, Nafion^®^ is of particular interest because it can be processed into thin transparent membranes that would be well suited to an optical sensor [27,32]. Nafion^®^ is an amorphous fluoropolymer which has refractive index values very similar to those water (slightly higher), thus rendering these materials transparent at macroscopic and microscopic levels of observation. The refractive index of Nafion^®^ film (*n* = 1.37 RIU at *λ* = 589.3 nm after an annealing process in a dry state), is lower than that of the fiber optic core (*n_c_* = 1.48 RIU), so light is partially reflected from the SiO_2_/Nafion^®^ interface. Nafion^®^ shows excellent optical transmission, even throughout multiple layers of the material. However, the refractive index of the hydrated film decreases and is even more similar to water. Figure 1b shows a schematic diagram of the fiber optic RH sensor based on Nafion^®^ thin film.

### 2.4. Characterisation of the Nafion^®^ Fiber Optic Sensing Structure

The manufactured Nafion^®^ thin films on fibers were characterized by several measurement methods. The layer thickness and its quality were tested by reflectance spectroscopy as well as by confocal microscopy and atomic force microscope (AFM, NT-MDT Spectrum Instruments, Moscow, Russia). Nafion^®^-based sensing structures were analyzed using a Ntegra Spectra commercial device (AFM Raman Confocal system; NT-MDT Spectrum Instruments, Moscow, Russia) in upright configuration. The surface optical uniformity and morphology of these Nafion^®^ structures was explored using an optical confocal microscope. The confocal laser scanning system consisted of a laser-scanning module with two lasers (532 nm and 633 nm). The pinhole size was adjusted to 100 µm. As confocal optical signal detector a photomultiplier tube was used. 

An AFM technique was used to assess morphology and roughness using the semi-contact topography mode. The surface roughness was calculated by dividing the integral of the surface of the pore/islands by the whole surface area given by AFM and characterized by the parameters surface average roughness (Sa) and root means square (Sq, RMS). The fabricated Nafion^®^ thin films were also characterized by optical microscopy. Prior to the optical test the whole Nafion^®^ structure deposited onto the end of a MM fiber optic, especially in the fiber core area, and the samples were placed in a special holder for a clearer observation of the Nafion^®^ surface and the Newton fringes. Digital image acquisition and analysis of the confocal and AFM sections were performed with NOVA software (NT-MDT Spectrum Instruments, Moscow, Russia).

### 2.5. Experimental Set-Up

**Figure 3:**
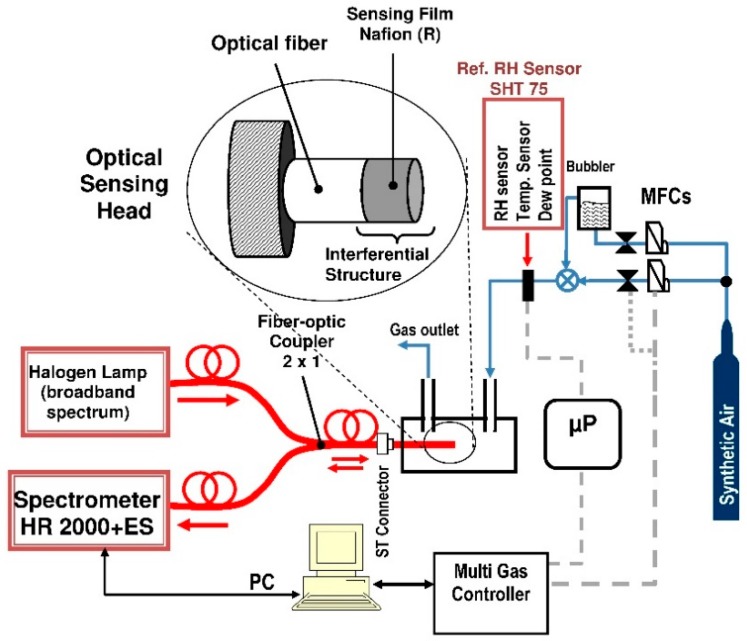
Schematic of experimental setup for RH measurements.

A diagram of the experimental setup, consisting of a halogen source, a multimode (MM) coupler, spectrometer and humidity sensors (FP probe and commercial SHT75, Sensirion AG, Staefa, Switzerland) is shown in Figure 3. The FP sensor probe is connected to the system by a ST/ST connector using liquid index-matching. The immersion liquid minimizes the reflection of light on a ST/ST connection. All kinds of optical fiber (lead-in fiber, 50/50 coupler, sensor probe) have similar outside diameters, which is in the order of 125 μm. Light from the halogen source (380–900 nm) is focused and transmitted via the lead-in fiber to the system. White light was guided into a fused silica fiber optic, and the reflected light was collected and analyzed using a spectrometer (HR 2000+ES; Ocean Optics, Largo, FL, USA). Light is partially reflected by the end face of the MM fiber covered Nafion^®^ film. Then the two reflections propagate back through the same fiber and generate interference fringes. 

For the sensor response examination, the sensing structure set in a PTFE/Viton holder was placed in a stainless steel flow chamber. Humid air was prepared by allowing passage of a dry air (synthetic air 79% N_2_, 21% O_2_; SIAD Poland, Ruda Śląska, Poland) from a cylinder through a deionized (DI) water bubbler. Humidity of the test gas prepared was balanced by mixing the humid air and dry (RH = 5.5%) air at a prescribed ratio of the flow rate (total flow rate: 500 sccm) and was monitored with a commercial humidity sensor (SHT75; Sensirion AG, Staefa, Switzerland). Multi-gas controller based on Bronkhorst mass flow controller-mixed gases with a precision exceeding 0.1%. All elements of the pneumatic circuit are made of stainless steel 316 L and PTFE. The idea of the measurements was to obtain the spectral reflection characteristics when exposed to a mixture of synthetic air with a varying volumetric content of water vapor.

### 2.6. Relative Humidity (RH) Measuring Methodology

A simple signal processing algorithm to estimate the optical response signal of FP humidity sensor probe based on the white-light interferometric fringe analysis approach has been proposed. The algorithm is based on analysis of the spectral interferograms in the linear regions—at the quadrature point (Q-point) [8,33]. Q-point of the FPI is in the middle of the interference fringe linear range, where the curve has the largest slope.

In this approach, the modified normalized interference spectrum of the FP sensor is measured at multiple wavelengths by using the measurement system given in Figure 1a as follows. First of all, without the connected a FP humidity sensor probe, the dark current of the spectral detector and the reference spectrum is measured. The reference spectrum is measured and recorded as reflected optical beam *I*_0_’(*λ*) from a clean fiber end (ST connector). The optical reflectance from the well-cleaved fiber end face was about *R*_0_ = 4% for fused silica fiber. Then, the fiber optic Nafion^®^-based FP humidity sensor probe is connected (ST/ST) and the reflected light power is measured *I*(*λ*). The modified reflectance of the Nafon^®^-based FP sensor probe *R_FP_*’(*λ*) is described by Equation (6):

(6) $$R_{FP}^{\prime }\left(\lambda \right)=\frac{I\left(\lambda \right)}{I_{0}^{\prime }\left(\lambda \right)}=R_{0}\left[R_{1}+R_{2}+2\sqrt{R_{1}R_{2}}\cos\left(\Delta \varphi \right)\right].$$

A simple method for humidity sensing based on analysis of the interferometric reflected spectral signal has thus been developed. The principle of the method is based on the resonant-wavelength-shift and intensity variation of reflectance measured at a fixed wavelengths close to Q-points the same fringe. The response time and recovery time of the sensor was defined as the time required to reach 90% and 10% of the total optical signal change for each humidity level, respectively.

## 3. Results and Discussion

### 3.1. The Analysis of Morphology of The Fiber Optic Sensing Structure

After fabrication, the FP sensor is tested for its morphology, uniformity, optical quality, sensitivity, repeatability and stability. Microidentity examinations of the multimode (MM) fiber optic covered Nafion^®^ thin film samples were performed using optical microscopy, confocal laser scanning microscopy (CLSM) and AFM techniques. The images of the Nafion^®^-based sensor probe are the optical fiber surface for the case of 2-layers. Thus, a sensor with fiber effective cavity length (*OPD*/2 = *nL*) of approximately 726 nm (±20 nm) has been used to characterize the polymer sensing film and to measure the RH of the air at room temperature (RT). The sensor has been optimized in the field of: multimode fiber optics application, light power source fluctuations (the intensity interrogation mode), the sensitivity and using VIS spectral range-thickness of the Nafion^®^ layer. No problems of adhesion of Nafion^®^ layers to fiber SiO_2_ substrates were observed. 

**Figure 4:**
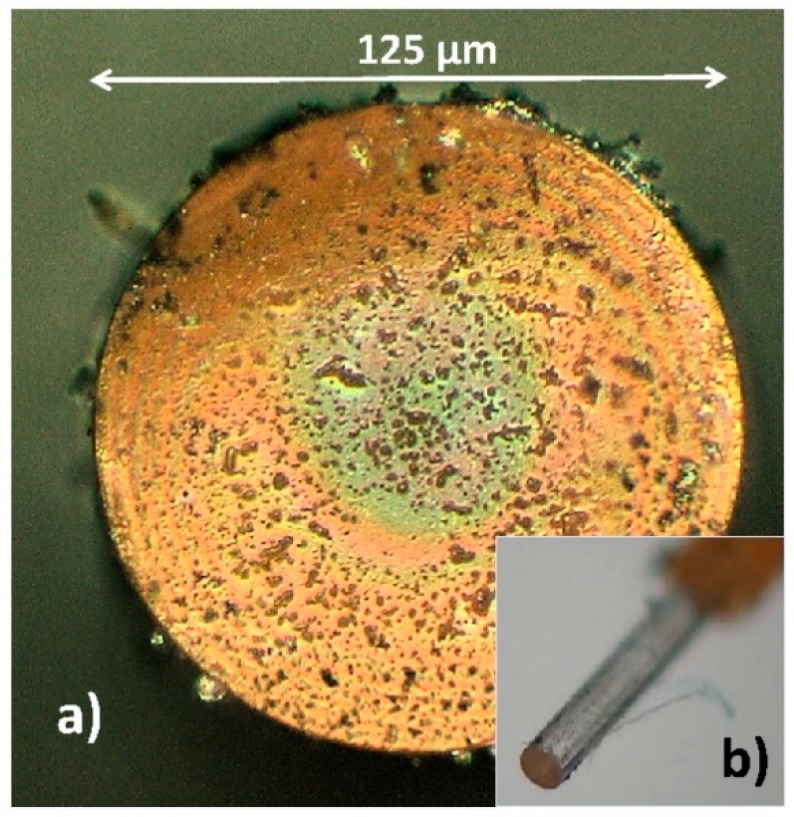
Micrograph of the Nafion^®^-based FP humidity sensor probe (**a**) end surface of FP fiber optic covered Nafion^®^ film, (**b**) fiber tip sensor.

First of all, an optical test of the whole Nafion^®^ structure deposited onto end of MM fiber optic, especially in the fiber core area, was done. Figure 4 shows typical micrographs of the Nafion^®^-based fiber optic RH sensor. Figure 4a presents a front view (fiber optic end surface) of the probe of the miniature humidity sensor as seen under an optical microscope and Figure 4b the whole tip of the sensor, respectively. From Figure 4 it can be seen that the end of the MM fiber was completely covered by Nafion^®^. These measurements indicate however a poor uniformity of the sensing structure. However, despite this fact Newton concentric rings in the fiber optic core region (center) are clearly visible. This means, as will be shown below, that the poor uniformity of the sensing structure is not critical for the interference effect obtained in this case. In order to better and more accurately assess the optical uniformity of structure, the quality of the structure being studied by CLSM and AFM technique simultaneously.

**Figure 5:**
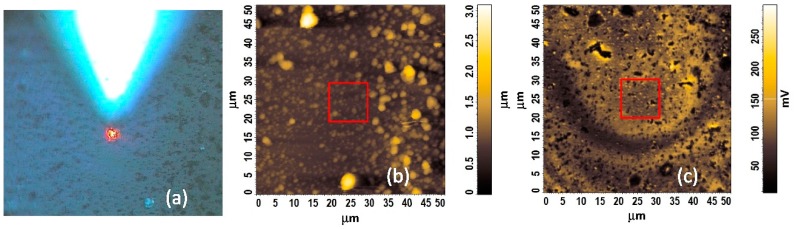
Surface analysis of the Nafion^®^-based fiber optic sensing microstructure; (**a**) optical micrograph with AFM tip position and laser (633 nm) spot (**b**) 50 µm × 50 µm AFM height topography image, (**c**) 50 µm × 50 µm CLSM image.

The fabrication of optical interferometric structures is a challenging task. The fringe pattern is produced when the effects of multiple reflected beams in the interferometer uniform cavity are highlighted. Undesirable difference optical paths can result from the thickness heterogeneity of the cavity, mirrors, and their local refractive index differences. Thus, an optical uniformity study of the layers is crucial to high-precision manufacturing of interferometric structures. Confocal microscopy is appropriate to check and study the optical uniformity of multi-layered structure interphases. Confocal Rayleigh imaging has been run simultaneously with AFM during one sample scan in the core region. All of the scanning probe experiments were carried out in ambient air environment with humidity RH = 44% at temperature of 28 °C. Figure 5 illustrates a fiber optic end surface of the RH probe in three imaging techniques. Figure 5a presents the Nafion^®^-based sensing structure of the humidity sensor under an optical microscope. In the field of view is the AFM tip VIT_P and laser spot (*λ* = 633 nm) on the surface of the sensing structure. Figure 5b,c shows the results of the scan of the surface RH sensor probe in the fiber optic core region (center) as topography image and optical properties image from 50 × 50 µm area, respectively. The Nafion^®^-based structure is transparent, thus, in Figure 5c the refractive-index gradient distribution in the MM fiber core can be observed. From the AFM and CLSM images of the whole MM fiber core area (50 × 50 µm), it can be seen that surface morphology of the Nafion^®^ has cluster-like structures, with a diameter in the range of several hundred nanometers. These low magnification images show that the Nafion^®^ film generally was uniformly coated onto end of the MM fiber. Therefore, further research was carried out on a smaller core area.

**Figure 6:**
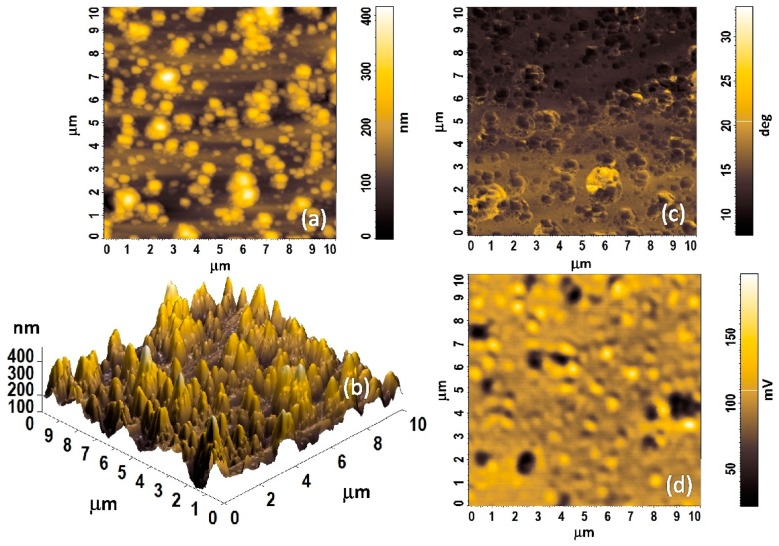
A top view AFM and CLSM images (area of 10 × 10 µm in the middle core of the fiber optic—red contour from Figure 5) of the Nafion^®^-based fiber optic sensing microstructure with 530 nm thickness. (**a**,**b**) 2D and 3D topographical images, respectively; (**c**) 2D AFM phase contrast image; (**d**) 2D CLSM image.

Figure 6a–d shows the comparison of AFM topography image (Figure 6a,b), AFM phase image (Figure 6c), and CLSM image (Figure 6d), respectively, of the same Nafion^®^ film in the MM fiber core region over a 10 × 10 µm area range (see Figure 5b,c red contour). The topography and phase contrast of Nafion^®^ thin films were investigated and visualized by means of the AFM semi-contact mode. The Nafion^®^ sensing structure roughness and porosity were characterized using AFM. Typical parameters of the Nafion^®^ thin film like pore size, roughness and porosity of different RH sensing structures were obtained through analysis of AFM 10 × 10 µm pictures using the NOVA software. The surface average roughness (*S*_a_) and surface roughness mean square (*RMS*/*S*_q_) of the typical films are about 60 and 80 nm, respectively, which demonstrates that the surface morphology is not quite smooth.

Cluster-like structures with a diameter of about 0.5–1 µm are clearly visible, particularly in the AFM phase images. These structures generally correspond to features in the topography image and CSLM image. The phase imaging is an AFM semi-contact technique. This method allows for imaging the distribution of changes in mechanical properties of the sample. Therefore in heterogeneous samples, for areas with different mechanical properties, can be observed as different phase angles. In addition, phase imaging improves the contrast and allows one to extract topography details. Nafion^®^ has ordered structures in which the hydrophilic groups are within a hydrophobic matrix composed of the fluorocarbon backbone of the polymer. The density contrast between the ionic clusters and the matrix gives rise to AFM phase contrast imaging [28,29]. The phase contrast between the hydrophilic sulphonic acid groups and the hydrophobic regions is strongly visible in Figure 6c and it should increase with humidity due to sorption of water into the hydrophilic regions [29].

### 3.2. The Analysis of Optical Properties and Humidity Sensing Performance

#### 3.2.1. Morphology Effects on the Optical Properties of Sensing Film

The preliminary results of Nafion^®^-based sensing interference structure were presented in [12]. This paper presents an extended analysis for a thinner 2-layer structure. The thinner structure has better sensitivity and faster response times.

**Figure 7:**
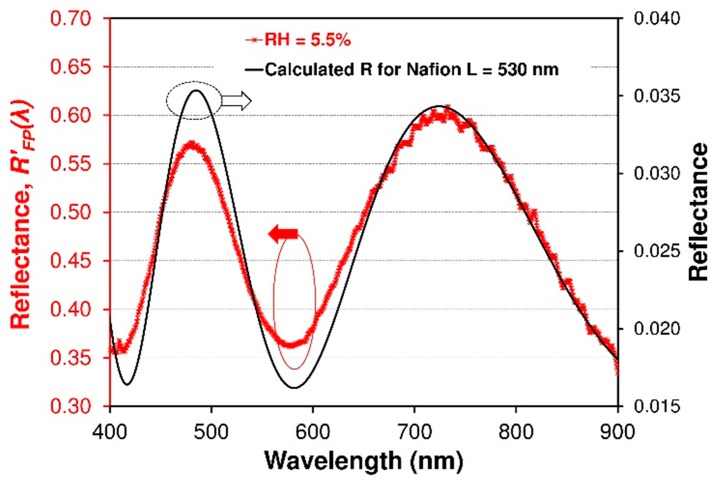
A comparison between measured normalized reflectance spectra (*R_FP_*’(*λ*)—red line) of the FPI sensor and a simulation (black solid line) of reflectance spectra with Nafion^®^ of 726 μm. Fringe patterns correlating to effective cavity length to *nL*, where *L* = 530 nm, *n* = 1.37 RIU.

**Figure 8:**
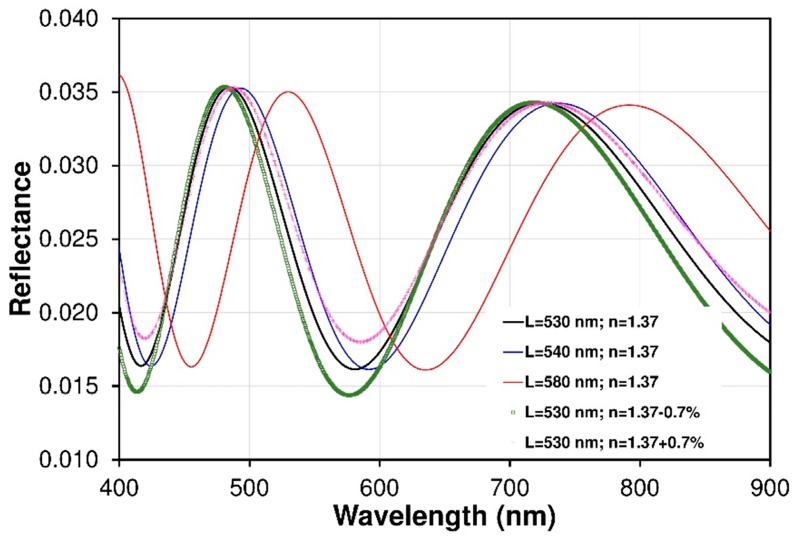
Comparison of the calculated interferograms for Nafion^®^ cavity with different patch length. The results show fringes shift effect and its contrast change due to cavity length and refractive index variation.

Nafion^®^ copolymers belong to the general class of segmented comb copolymer materials and generally consist of a hydrophobic fluorocarbon backbone with hydrophilic sulfonic acid side-chains. Most early studies [27,28,29,30,31] as well as this research have shown the heterogeneity of Nafion^®^ films in both their morphology and optical properties due to the complex roles of chemical structure and intramolecular interactions. Nafion^®^ exhibits strong morphologically heterogeneous, possessing domains with varying degrees of order and non-uniform grain boundaries, particularly in bulk films and membranes [27,28,29]. As the Nafion^®^ thickness decreases, changes in the behaviour of the material (e.g., changes in swelling process) can be expected due to a disordering of the film hydrophilic/hydrophobic structure on the molecular and nanometer scale. Given these facts, optical and different information from bulk films is then an average of this large heterogeneity, and may be different than for size-limited structures. Nevertheless, despite this heterogeneity, the reflected optical signal is averaged and the interference signal is clearly visible. Figure 7 shows fringe patterns correlating to cavity length (*L*) about of 530 nm (thickness of the Nafion^®^ thin film). This covered optical fiber is clearly suitable for Fabry-Perot construction as it demonstrates Fabry-Perot fringes. The theoretical spectral reflectance of the interferometric structures was modelled by means of software developed in the LabVlEW™ environment. This model is based on Fresnel’s theory and the matrix formalism of the propagation of electromagnetic waves thorough layered structures. The investigations concerned the Vis-NIR range of spectra (350–900 nm) in the range of ±25 degrees the angle of non-polarized light incidence on the structure. The model does not take into account the intermodal interferences in the MM fiber. The results of numerical simulation presented in Figure 7 (black solid line) illustrate the excitation of interference phenomena in the structure as a function of the wavelength. Figure 8 shows a comparison of the calculated interferograms for the Nafion^®^ cavity with different path length. The results show fringe shift effects and its contrast change due to cavity length and refractive index variation. This modeling allows me to assess the effect of parameters on the final fringe pattern of the FPI sensor.

The modified normalized interference spectrum of the fiber optic sensor at multiple wavelengths is measured by using the measurement system given in Figure 3. To investigate the performance of the tested sensor, the sensitivity, working range, linearity, reversibility and response and recovery times of the sensor are all analyzed.

#### 3.2.2. Resonant-Wavelength-Based Humidity Sensing

**Figure 9:**
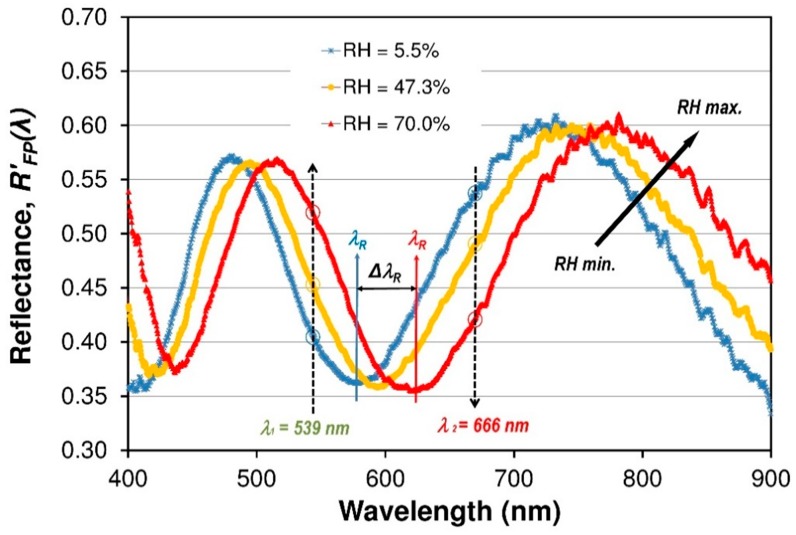
Normalized spectral reflectance signals (*R_FP_*’(*λ*)) of the FPI sensor at three operating RH of the air at RT (22 °C). Device presents a non-linear tuning fringe pattern for RH variation between 5.5% and 70%.

When the RH in the gas flow chamber was increased and then decreased in the RH range of 5.5%–80.0% at 22 °C room temperature (RT), the reflected spectra *R_FP_*’(*λ*) of the FP fiber optic sensor were measured for the RH increase and for the RH decrease. To measure the performance of the FP sensor, the evolutions of the modified reflectance spectra of the fiber optic sensor with the RH change measured by the fiber optic spectrometer as shown in Figure 9, which gives the experimental results for reflectance *R_FP_*’(*λ*) and a comparison of the results for three different humidity levels (5.5%, 47.3%, and 70.0% RH, respectively). It can be seen that the minima and maxima of resonance relationships, which is caused by interfering light beam in the Nafion^®^ thin film will shift to a longer wavelength as the RH increases because the water molecules will swell the Nafion^®^ film and thus mainly increase the thickness of the sensor film, as was discussed above. 

**Figure 10:**
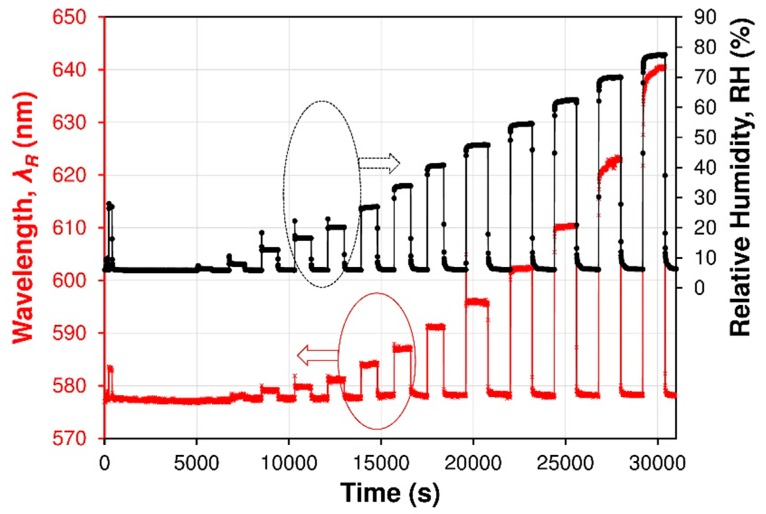
Spectral response (resonant wavelength *λ_R_* shift) of the Nafion^®^-based fiber optic device used as FPI sensor (red line) and of a commercial SHT75 sensor (black dotted line). The FPI sensor device was set near the commercial sensor in the measurement flow cell, where the humidity was changed with time.

**Figure 11:**
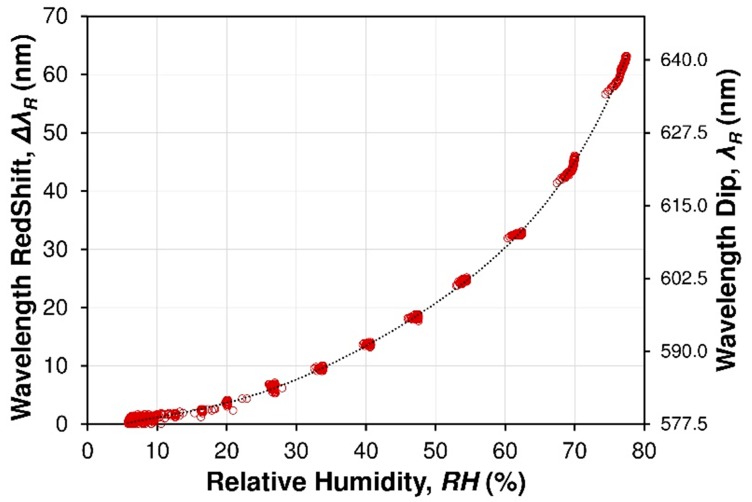
Resonant wavelength *λ_R_* and wavelength red shift Δ*λ_R_* of the dark fringe (central spectra region from Figure 9) vs RH. Points (red) are the experimental data; solid line (black): calibration curve given by fitting. Experimental reference measurements of RH% detection through the commercial SHT75 humidity sensor.

To investigate the dynamic performance and sensitivity of the FP sensor, the RH in the gas flow chamber is set to switch between RH_min_ 5.5% and several humidity levels up to RH_max_ 80% at RT. The RH level was monitored with a commercial humidity electronic sensor in the same chamber. Some cycles of the RH switching are measured and shown in Figure 10. The wavelength shift of the resonant dark fringe (the fringe is chosen arbitrarily) in the wavelength range of 530–680 nm was traced with the increase and decrease in RH. The measured wavelength redshift with the RH variation is shown in Figure 10. It can be seen that the chosen dark fringe, which is caused by destructive interference of light beam in cavity, will redshift from 576 nm at 5.5% RH to 640 nm at 78% RH. It can be observed in Figure 11 that the fiber optic sensor exhibits a very non-linear performance and sensor has a short response and a very good reversibility without any hysteresis.

#### 3.2.3. Intensity-Based Humidity Sensing

**Figure 12:**
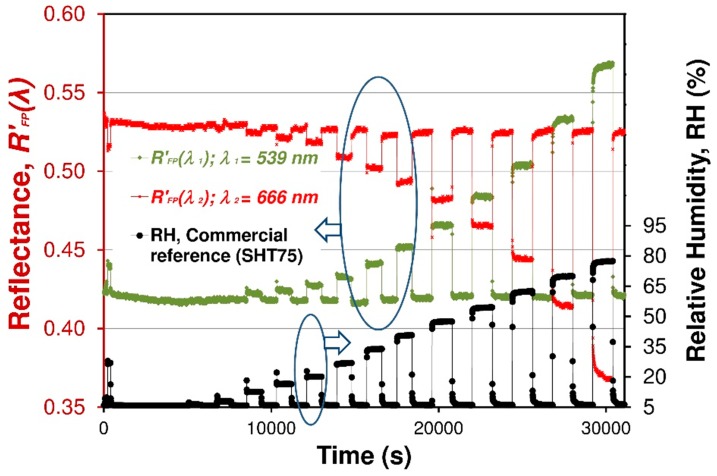
Normalized reflectance signals (*R_FP_*’(*λ*)) (at *λ*_1_ = 539 nm—green and *λ*_2_ = 666 nm—red) vs. time of the FPI sensor. For comparison, those raw measured optical signals are presented with response of a commercial SHT75 sensor (black line).

The sensor is interrogated by spectral-domain white-light interferometry. In practical applications, it is more desirable to sense RH based on intensity variation since only an optical power meter instead of spectrometer is required to sense the RH, because the working mode can largely reduce the application cost of the sensor. The principle of intensity-based RH sensing is shown in Figure 9. To investigate the performance of intensity-based RH sensing, an optical signal response the reflectance *R_FP_*’(*λ*) at a fixed wavelengths are measured. Figure 12 illustrates the dynamic optical response for fiber optic FPI Nafion^®^-based RH sensor versus different RH values. Humidity was increased from about 5.5% RH to 80% RH at room temperature. The optical signals *R_FP_*’(*λ*) for two different wavelengths of light beam (666 and 539 nm) are presented. In this case, the two wavelengths can be chosen arbitrarily due to the nature and shape of the interferometric fringe pattern of the structure.

Those two measured wavelengths are near to each other in the spectrum (in the two linear regions of the same fringe); therefore, the output signal is generally insensitive to bending losses [27]. The result of that division was the response *S* of the sensor and this result has been presented. The response magnitude *S* of the sensor was defined as Equation (7):

(7) $$S=\frac{R_{FP}^{\prime }\left(\lambda_{1}\right)}{R_{FP}^{\prime }\left(\lambda_{2}\right)}.$$

**Figure 13:**
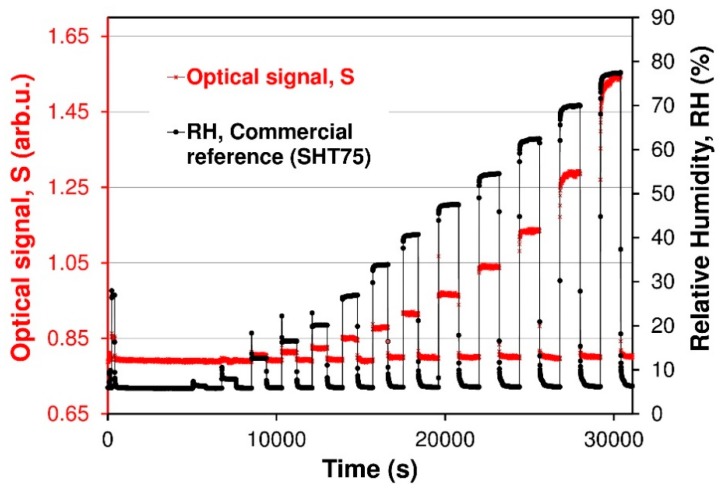
Optical response signal *S* vs. time of FPI sensor.

Extracting data from spectra interferogram (see Figure 10) according to the procedure described in Section 2.6 and above correspondingly, a relationship between the relative output optical signal *S* of FPI fiber optic sensor and RH can be obtained as shown in Figure 13. It is also possible to measure the color coordinates of the interference color of sensing structure. This color measurement can be accomplished by using simple color sensors such as TCS3200 (TAOS Inc., Plano, TX, USA). An example of such a measure of the interference color is shown in [30].

**Figure 14:**
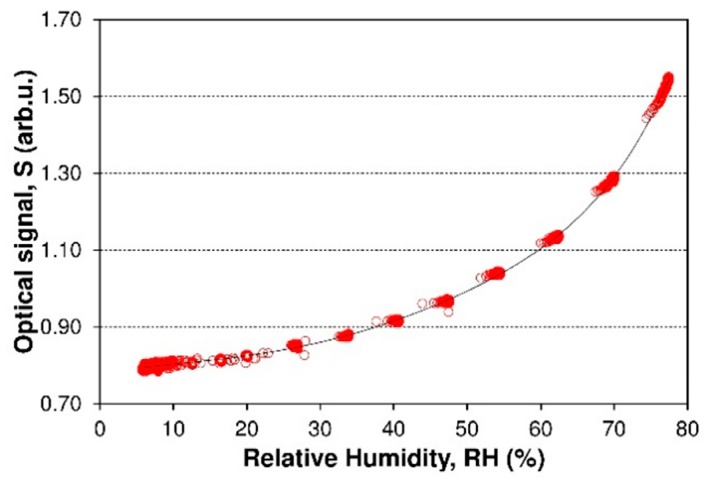
The calibration curve *S* = *f*(*RH*) of FPI sensor. The red measurement points show data, and the black solid line shows a non-linear fit for data.

A few cycles of the RH switching are measured and shown in Figure 13. It can be seen that the optical signal *S* increased by 0.75 as the RH increased from 5.5% to 78% and holds as the RH recorded by a commercial sensor, which indicates that the fiber optic FPI sensor has a good repeatability and a good reversibility without any hysteresis. The output optical signal *S* increases nonlinearly from 0.80 to 1.55 as RH increases from 5.5% to 80% in the chamber. Likewise the output optical *S* decreases as RH descends back to 6%. Figure 14 shows the nonlinear relationship between the measured normalized optical signal *S* and the RH of the FPI sensor.

**Figure 15:**
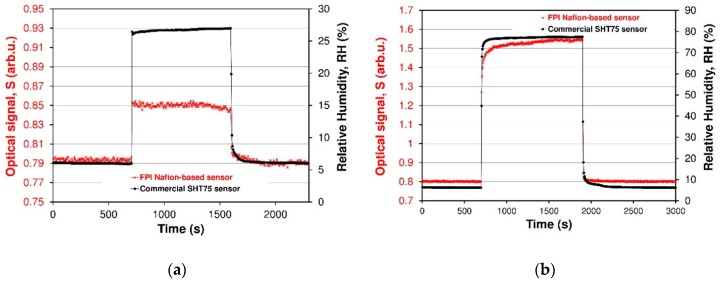
Calculation of response and recovery times from dynamic response graphs in Figure 12. The curves are relative to a RH of (**a**) 27% and (**b**) 78%, respectively.

As a result of investigations of dynamic behavior of the sensor, response and recovery times were calculated. The data were collected in Table 1. The response and recovery time of the FPI sensor has been found to depend on RH level. It is evident from Figure 13 that FPI sensors possesses similar responses and recovery time values better than 5 s for RH < 50%, whereas the response time becomes longer than the regeneration time for higher humidity RH > 50%. Figure 15a,b is a zoom-in of a RH 27% and 78% cycle, respectively. From Figure 15a, it can be seen that the optical signal S increases by 0.06 when the RH is varied from 5.5% to 27% and comes back to the original S for RH 5.5%. From Figure 15a, it can be seen that the response time for the FPI sensor is less than 5 s and the recovery time is also better than 5 s. Response and recovery times of sensors affect the RH increasing and RH decreasing processes with RH ranging from 5.5% to 78% (Figure 15b). The sensor response time (humidification from 6% to 78% RH) was about 80 s, and the recovery time (desiccation from 78% to 6% RH) was about 12 s. The recovery times for higher humidity levels were better than recovery times of reference capacitive sensor. The response time is a little slower than that of the about 16 s (RH 78%, SHT 75) is because the unique (more complex) water molecules sorption processes by Nafion^®^ polymer films.

The response times measured in the experimental setup with FPI sensor were comparable to those of RH fiber sensors based on etched POF structures [6,7]. The etched POFs [6,7] were proposed for fiber optic humidity sensors with one of the lowest response times for RH sensors. Rajan et al. [6] presented a POFBG RH sensor with a 4.5 s response time for 30% RH variation, whereas Leal-Junior et al. [7] show etched stress-POF sensors with response times lower than 2.0 s for 10% RH variation. It should be noted that the response times recorded for low humidity levels (below 40% RH at RT) are limited by the time resolution of the measurements, which is 5 s in this study. The FPI sensor obtains full signal saturation after 5 s. Future studies assume more accurate testing of sensor dynamic parameters.

The water molecule interactions of polymer Nafion^®^ membranes are of significant interest when these materials are used in, for example, fuel cells [25,26,27,28,29,30,31,32]. Water and water vapor sorption and transport in Nafion^®^ has been extensively studied in recent years by several different methods [27,28,29,30,31]. The results of those investigations have indicated differences in the diffusivity of water. This has been discussed by a great number of authors in the literature [27,30]. A series of recent studies has indicated that water absorption and transport in and through Nafion^®^ membranes is not simply a diffusion process, but interfacial mass transport across the gas/film interface presents a major resistance to water transport. Water absorption is described by interfacial mass transport that controls water absorption, at early timepoints. At longer times, water absorption is controlled by the dynamics of polymer swelling and relaxation. The thin film must swell to accommodate the volume change when the water molecule coordinates with a sulfonic acid site in the Nafion^®^. Studies of the water sorption by Nafion^®^ indicate that the time for water uptake scales with length which is consistent with the shrinking-core model for water uptake. Water is sorbed at the gas/film interface, diffuses through a shell of swollen membrane and then reaction occurs at the core where water causes the Nafion^®^ film to swell [27,28,29,30]. These studies have shown that Nafion^®^ thin film shrinks for the water molecules to leave the swollen film. However, the bulk membrane of Nafion^®^ does not always need to shrink. The literature review shows that water desorption from a water molecules strongly saturated Nafion^®^ films and membranes was an order of magnitude faster than water sorption. It these optical studies confirm the current knowledge of water sorption processes by Nafion^®^ polymer membranes.

As shown above, good results were obtained with this Nafion^®^-based RH active optrode and simple technological method. Nevertheless, the usage of this sensor is limited to the detection of relative humidity level below condensation process conditions. Water condensing on a surface of a Nafion^®^ layer changes the conditions for the interference of light waves (change *R*_2_ due to change of dielectric contrast from Nafion^®^/air to Nafion^®^/water). Because of this potential limitation, the sensor can be treated as an inexpensive, small and requiring very little power for operation, alternative to electronic RH sensors.

The intensity interrogation method uses a broadband light source and provides relative intensity measurements without requiring wavelength information. The relative (signal) power measured from the two Q-points (two slopes, two wavelengths) of the same fringe allow one to eliminate the effect of input power fluctuation. It was assumed that source power fluctuations are comparable in the narrow spectral range (the spectral range of the analyzed fringe) and the spectral characteristic of the source do not change significantly. Thus, this method has a large tolerance to the power stability of the light source.

Regardless of the results, future research will focus on explore temperature dependences of Nafion^®^ cavity. The Nafion^®^ layer will also deform due to the thermal expansion, but, experimental works show the Nafion^®^ thermal coefficient strongly depends on the thickness of the layer and the type of substrate. The literature [44,45] indicates that for the annealed Nafion^®^ layers on SiO_2_ substrates, the relative thickness changes ΔL/L do not exceed 2% for temperature changes of ΔT = 50 °C in the low temperature range of 20–70 °C. The influence of temperature on the Nafion^®^’s refractive index is even less important [45]. The refractive index of Nafion^®^ after absorption of water vapor decreases to a max. of about 0.7%. The results also demonstrate that the changes in volume caused by differences in moisture content will have a much greater impact than temperature (and other factors) on the Nafion^®^ layer. Differences depending on the type of polymer macromolecule are small. The change in temperature mainly affects the polymer matrix-polytetrafluoroethylene (PTFE). Thermal expansion tests and the influence of temperature on the index of refraction were presented in [45].

Further studies should also investigate the limitations of sensor operation in harsh and chemical environments with or without polar and hydrophilic analytes at different temperatures. Nafion^®^ is sensitive to polar components such as alcohol vapors, ammonia and other chemicals. Ammonia has an influence on changing the refractive index of the Nafion^®^ layer in the absence of water vapor. In the presence of humid atmosphere, ammonia interacts with water reduces the swelling of Nafion^®^. Research on the interaction of ammonia with Nafion^®^ has been presented in the literature [40]. The influence of alcohol vapors is the same as the effect of water vapor. Therefore, selectivity for this type of chemical compounds should not be expected.

## 4. Conclusions

Using Nafion^®^ films as humidity sensing materials, here a microscale fiber optic humidity sensor was fabricated. The fiber-based intrinsic Fabry-Perot interferometric sensor for air humidity measurements has been demonstrated. The sensor is extremely simple and small. Fringes were readily generated by use of a Nafion^®^ thin film as the resonant cavity of the interferometer. This thin film acts as a sensing layer. Both the sensor and the signal processing algorithm work well, showing the system’s capability of operating at wide range of RH of the air. A prototype fiber optic sensor was tested from 5.5% RH to 80% RH at temperature of 22 °C with excellent reversibility. This humidity sensor shows a fast response and recovery time (comparable to the conventional reference capacitive sensor). For humidity levels in the RH range of 5.5%–80.0%, the sensor’s response and recovery times were found to be about from 5 to 80 s and from 5 to 12 s, respectively. Therefore, FPI sensor appears to be an ideal proposition for humidity sensing with high sensitivity for widespread applications requiring small sensors without electrical power in measurement environment.

## Figures and Tables

**Table 1:** Response and recovery times of humidity FPI sensor.

Response Time, t_90_ (s)		Recovery Time, t_10_ (s)	
RH 6% → 27%	RH 6% → 78%	RH 27% → 6%	RH 78% → 6%
<5	80	<5	12
